# Rapid molecular diagnostic tests fail to PERFORM in febrile children

**DOI:** 10.1016/j.lanepe.2023.100707

**Published:** 2023-08-04

**Authors:** Shamez N. Ladhani

**Affiliations:** St. George’s University of London, Cranmer Terrace, London SW17 0RE, UK

Fever remains one of the most common reasons for children seeking healthcare. At the same time, identifying the small minority with a serious underlying bacterial infection from the vast majority who suffer from self-limiting viral infections remains one of the most challenging tasks for clinicians assessing children in the Emergency Department. This task is further compounded by the highly successful childhood immunisation programmes that have significantly reduced the burden of serious bacterial infections in children.^1^ In high-income countries with established national immunisation programmes, bacterial infections have become so uncommon in children that earlier “rule-in” algorithms are now being replaced with strategies to “rule-out” serious bacterial infections and allow children to be discharged home safely with appropriate safety-netting advice.^2^ The development and increasing availability of molecular diagnostic tests that can rapidly detect bacteria, viruses, fungi and parasites, therefore, have the potential to diagnose infections rapidly, allowing for more informed decision-making on the risks of underlying serious infection and the need for invasive investigations, hospital admission and empiric antimicrobial therapy.

In this issue, researchers in 17 hospitals across 9 European countries prospectively recruited febrile children aged <18 years from emergency departments, inpatient wards and intensive care units as part of an EU-funded multi-centre study, PERFORM (www.perform2020.org).^3^ In addition to detailed clinical, laboratory and imaging data, the researchers performed centralized molecular testing for 25 viral, 27 bacterial and 6 fungal targets on all available throat swabs and blood samples from 4611 patients and 1061 controls.

The most important finding of this study is that, despite best practice investigations at each participating hospital, the aetiology of infection remained unknown in 75% (3477/4611) of the febrile children, with the remaining classified as either definite bacterial (14%, n = 643) or definite viral (11%, n = 491) infection. Additionally, centralised molecular testing for 19 respiratory and 27 blood pathogens added only 2% to assignment of cases as definite bacterial infection and only 1% to definite viral infection. These findings highlight the limitations of current diagnostic tests and emphasise the value of clinical judgement and experience in distinguishing children with serious bacterial infections who may require hospital admission, supportive treatment and/or antibiotics from those with self-limiting viral infections who can be safely discharged home without additional interventions.

Another important finding of this study is that identification of a virus in a febrile child did not exclude an underlying bacterial infection. Moreover, although molecular testing increased the number of patients with detected viruses, they did not significantly improve diagnostic assignment because viruses were also identified in a high proportion of febrile children with confirmed or probable bacterial infections. Local diagnostic tests and central molecular tests, respectively, detected viruses in 360 (56%) and 161 (25%) of children with definite bacterial infection. At the same time, only three respiratory viruses were detected more frequently in children with definite viral compared to definite bacterial infections: influenza A, influenza B and respiratory syncytial virus (RSV). Consequently, identification of a virus had poor predictive values of 0.64 and 0.68 for excluding definite or probable bacterial infection, respectively, again highlighting the value of clinician judgement and experience in identifying, managing and treating seriously unwell children, even in the context of a confirmed viral infection.

The findings of this study highlight important messages in terms of both inefficient healthcare utilisation associated with unnecessary hospitalisations and contributing to the global problem of antibiotic overuse. For example, 80% of all patients, including 71% of those with confirmed and 48% of those with probable viral infection, were prescribed antibiotics by the medical teams responsible for their care. The complex interplay between viruses and bacteria—which we still poorly understand—raises other problems. For example, even if we develop large-scale multiplex viral diagnostic tests, their value in clinical practice will be limited if we cannot identify the small proportion of children with concurrent bacterial infections. At the same time, 22% (105/485) of the control cohort in this study had at least one virus detected on a throat swab, raising further questions on the clinical utility, time, costs and resources needed for routine use of multiplex viral diagnostic tests in healthcare settings.

The researchers of this large-scale, multi-country study rightly conclude that currently available molecular tests do not help categorise children as having a self-limiting viral or a serious bacterial infection, raising important questions about the clinical value of individual pathogen detection test. There are, however, other exciting novel approaches being developed to discriminate bacterial from viral infections, such as host-RNA signatures and combination biomarkers, which are promising.^4^^,^^5^ Whether such complex tests can be scaled down for routine use in hospital settings—ideally as rapid point-of-care (POC) tests in the Emergency Department—remains to be seen.^6^ All in all, the PERFORM consortium should be commended for their excellent work that has resulted in multiple high-quality peer-reviewed academic publications in the field of paediatric infectious diseases, diagnostics and antimicrobial stewardship, initially through ILULU (https://www.diamonds2020.eu/our-research-history/ilulu/) and EUCLIDS (https://www.diamonds2020.eu/our-research-history/euclids/), then through PERFORM (https://www.perform2020.org/media/publications) and, more recently, through DIAMONDS (https://www.diamonds2020.eu/). Until we have better rapid diagnostic tests, however, we need to continue to train, support and, most importantly, acknowledge the difficult tasks faced by frontline healthcare professionals who have to constantly rely on their clinical skills and experience to identify the small minority of seriously ill children from the large number of febrile children with self-limiting viral infections who seek medical care every single day.

## Declaration of interests

None.
